# Prediction of the axial compression capacity of ECC-CES columns using adaptive sampling and machine learning techniques

**DOI:** 10.1038/s41598-025-86274-7

**Published:** 2025-02-04

**Authors:** Khaled Megahed

**Affiliations:** Department of Structural Engineering, Mansoura University, PO Box 35516, Mansoura, Egypt

**Keywords:** Machine learning, Engineered cementitious composites, Adaptive sampling, Finite element modelling, Symbolic regression, CatBoost model, Civil engineering, Computer science, Scientific data, Statistics

## Abstract

An innovative form of concrete-encased steel (CES) composite columns incorporating engineered cementitious composites (ECC) confinement (ECC-CES) has recently been introduced, displaying superior performance in failure behavior, ductility, and toughness compared to traditional CES columns. This study presents an innovative approach to predicting the axial capacity of ECC-CES columns using adaptive sampling and machine learning (ML) techniques. This study initially introduces a finite element (FE) modeling for ECC-CES columns, integrating material and geometric nonlinearities to accurately capture the inelastic behavior of ECC and steel through appropriate constitutive material laws. The FE model was validated against experimental data and demonstrated strong predictive accuracy. An adaptive sampling process is employed for efficient exploration of the design space to generate a database of 840 FE models. Subsequently, seven ML models are utilized to predict the axial compression capacity based on the FE database. These models were comprehensively evaluated, displaying a superior prediction performance compared to design standards such as EC4 and AISC360. From evolution metrics, the Gaussian process regression, CatBoost (CATB), and LightGBM (LGBM) models emerged as the most accurate and reliable model, with nearly more than 97% of FE samples within the 10% error range. Despite the robust performance of the ML models, their black-box nature limits practical applicability in design contexts. To address this, the study proposes a symbolic regression-derived design that offers interpretable, explicit design equations with competitive performance metrics.

## Introduction

Concrete-encased steel composite (CES) columns are widely preferred in high-strength structural applications such as high-rise buildings and bridges due to their exceptional strength, stiffness, and ductility. These columns offer enhanced structural efficiency by combining the strength of steel with the durability of concrete, resulting in a more compact and lightweight cross-section compared to conventional reinforced concrete (RC) columns. The concrete encasement also provides additional benefits, such as increased fire and corrosion resistance, as well as delaying the onset of local buckling in the steel section, which further extends the lifespan of the column^1,2^. While traditionally, CES columns have been constructed using normal-strength concrete, recent research has focused on the potential of high-strength concrete (HSC) to further improve the performance of these columns^1–3^. Although HSC offers advantages such as increased strength and stiffness, it also introduces issues like premature spalling and brittle failure under compression, which can reduce both the load-bearing capacity^4,5^ and durability of the columns^6–8^.

In response to these challenges, a novel approach has been developed by using engineered cementitious composites (ECC) to encase CES columns, which helps mitigate issues associated with HSC, such as explosive spalling and brittle failure^4,5,9^. ECC is a highly ductile material, exhibiting significantly higher tensile (ε ≈ 0.4 to 3%) and compressive (ε ≈ 0.38–0.6%) ductility compared to conventional concrete^4,5,10^. This enhanced ductility allows ECC to better control cracking and spalling, particularly under high compressive loads, which leads to improved compressive performance in ECC-concrete encased steel (ECC-CES) composite columns^4,5,9^. Studies have demonstrated that using ECC not only increases the structural reliability of CES columns but also improves their ability to resist sudden failure modes, thus offering a more robust solution for high-performance composite columns.

Due to the novelty of ECC-CES columns, there is limited literature on both numerical and analytical models to predict their compressive behavior. To address this gap, a finite element (FE) model has been developed using ABAQUS^11^, which can accurately predict the compressive response of ECC-CES columns. The FE model incorporates detailed material properties for each component of the column, appropriate mesh sizes, geometric imperfections, and contact interface properties to capture the complex inelastic behavior of the composite columns. The model has been validated against previous experimental results, showing good agreement in terms of initial stiffness, ultimate strength, and load-deformation responses^10^. This validated model provides a reliable framework for further investigations into the structural behavior of ECC-CES columns, enabling researchers to explore various design configurations and material properties without the need for extensive experimental testing.

To further enhance the efficiency of FE models in predicting complex behaviors, surrogate models, also known as meta-models, are often employed. Surrogate models offer simplified representations of computationally expensive simulations, enabling faster results while maintaining reasonable accuracy. Common surrogate modeling techniques include polynomial regression surfaces^12^, Kriging models or Gaussian models^13,14^, neural networks^15^, and support vector regression^16^. These models have been successfully applied in various fields, such as analyzing the behavior of rockfill dams using polynomial chaos expansion and deep learning networks^17^.

After generating a finite element (FE) dataset using the adaptive sampling strategy, this data can be effectively employed with machine learning (ML) techniques to predict the axial capacity of composite columns. ML has emerged as a powerful tool in addressing complex structural engineering problems, providing accurate predictions while significantly conserving experimental and FE resources. By utilizing existing FE datasets, ML reduces the need for extensive FE modeling, allowing for faster and more efficient model development. For example, Tran et al.^18^ utilized a database of 300 samples obtained from uniaxial loading tests to train ML models designed to predict the axial strength of square concrete-filled steel tube (CFST) columns. The ML models provided reliable predictions, effectively reducing the reliance on additional experimental data. Similarly, Zarringol et al.^19^ utilized a larger experimental dataset of 3091 CFST columns with varying geometries, including rectangular and circular columns with and without eccentricity. This extensive database was employed to train ML models to predict the axial behavior of CFST columns under different loading conditions. Furthermore, Hou and Zhou^20^ focused on optimizing various ML models to enhance the predictive accuracy for both stub and long circular CFST columns. Their work involved using a variety of ML techniques, including backpropagation artificial neural networks (ANN), Gaussian process regression (GPR), genetic algorithms, radial basis function neural networks (RBFNN), and multiple linear regression (MLR) models. These optimized models were shown to predict the axial compressive strength of composite columns with high precision, further demonstrating the potential of ML in predicting complex structural behaviors.

## Finite element modelling

Accurate prediction of structural member behavior depends heavily on the use of appropriate material properties^3^. To enhance both strength and ductility, researchers have developed hybrid fiber ECCs by combining low-modulus fibers such as polyvinyl alcohol (PVA) with high-modulus fibers like steel within a high-strength matrix^5,9,21^. The addition of steel fibers significantly improves ECC fire resistance while effectively reducing crack widths, which in turn lowers permeability and boosts long-term durability. Consequently, steel-PVA hybrid fiber ECC provides superior crack resistance, enhanced damage control, greater compressive toughness, and improved energy dissipation compared to traditional concrete.

The compressive stress–strain behavior for ECC can be described using a non-linear function for the pre-peak ascending portion^21^ and a bilinear relationship for the post-peak descending portion^22,23^ as follows:

1 $$f_{Ec} = \left\{ {\begin{array}{*{20}l} {f_{c}^{\prime} \left[ {\frac{\varepsilon }{{\varepsilon_{c}^{\prime} }}\left\{ {\alpha_{2} \left( {1 - \frac{\varepsilon }{{\varepsilon_{c}^{\prime} }}} \right) + 1} \right\}} \right]} \hfill & {0 < \varepsilon \le \varepsilon_{c}^{\prime} } \hfill \\ {f_{c}^{\prime} + \left( {f_{cu} - f_{c}^{\prime} } \right)\left( {\frac{{\varepsilon - \varepsilon_{c}^{\prime} }}{{\varepsilon_{cu} - \varepsilon_{c}^{\prime} }}} \right)} \hfill & {\varepsilon_{c}^{\prime} < \varepsilon \le \varepsilon_{cu} } \hfill \\ {f_{cu} + \left( {f_{cf} - f_{cu} } \right)\left( {\frac{{\varepsilon - \varepsilon_{cu} }}{{\varepsilon_{cf} - \varepsilon_{cu} }}} \right)} \hfill & {\varepsilon_{cu} < \varepsilon \le \varepsilon_{cf} } \hfill \\ \end{array} } \right.$$

**Fig. 1:**
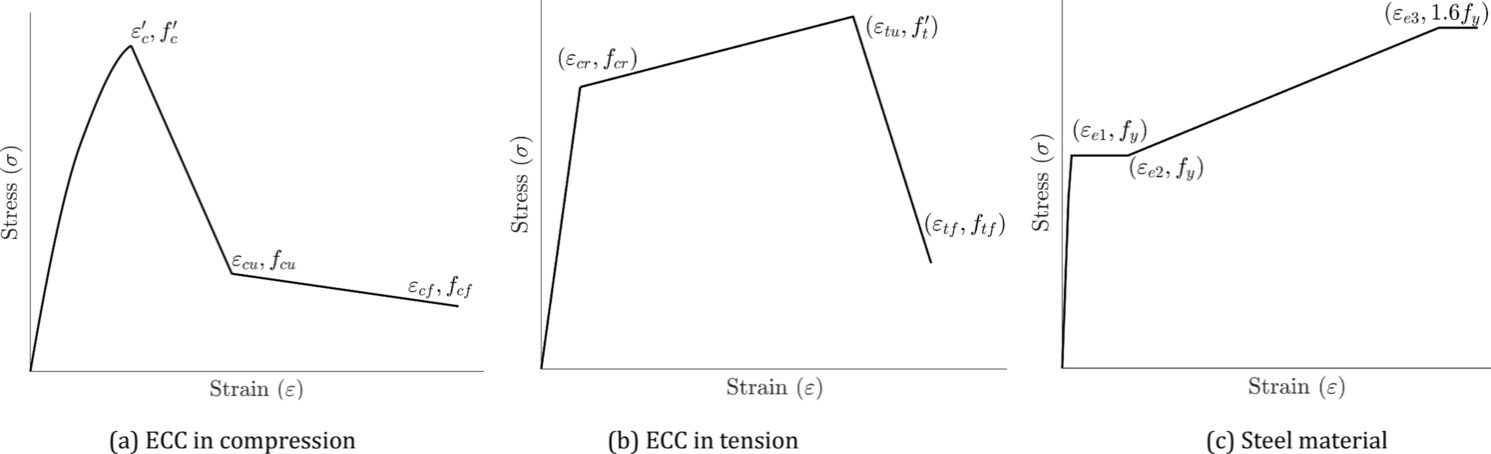
Stress–strain curves for materials used in finite element modeling.

Here, ${f}_{c}^{\prime}$, ${f}_{cu}$ and ${f}_{cf}$ represent the peak, ultimate and failure compressive strengths of ECC, respectively, while ${\varepsilon }_{c}^{\prime}$, ${\varepsilon }_{cu}$ and ${\varepsilon }_{cf}$ are their corresponding strains (Fig. 1a). The parameter ${\alpha }_{2}$ defines the shape ascending part^21^ and is calculated as:

2 $${\alpha }_{2}=\frac{\left({E}_{i}{\varepsilon }_{c}^{\prime}/{f}_{c}^{\prime}\right)-1}{1-\left(0.35{f}_{c}^{\prime}/{E}_{i}{\varepsilon }_{c}^{\prime}\right)}$$

where ${E}_{i}$ is the initial tangent modulus. The values of ${f}_{cu}$ and ${f}_{cf}$ are taken as 0.25 ${f}_{c}^{\prime}$ and 0.15 ${f}_{c}^{\prime}$, respectively, while ${\varepsilon }_{c}^{\prime}$, ${\varepsilon }_{cu}$, ${\varepsilon }_{cf}$ are assumed to be 0.5%, 1.5 ${\varepsilon }_{c}^{\prime}$ and 5 ${\varepsilon }_{c}^{\prime}$, respectively^24^.

The tensile behavior of ECC in this study is described by a trilinear model (Eq. (3))^23,25^. It assumes a linear increase in stress with strain up to the first cracking point, followed by pseudo strain-hardening up to the ultimate point, and a final drop to the failure point.

3 $$f_{Et} = \left\{ {\begin{array}{*{20}l} {E_{i} \varepsilon } \hfill & {0 < \varepsilon \le \varepsilon_{cr} } \hfill \\ {f_{cr} + \left( {f_{t}^{\prime} - f_{cr} } \right)\left( {\frac{{\varepsilon - \varepsilon_{cr} }}{{\varepsilon_{tu} - \varepsilon_{cr} }}} \right)} \hfill & {\varepsilon_{cr} \le \varepsilon \le \varepsilon_{tu} } \hfill \\ {f_{t}^{\prime} + \left( {f_{tf} - f_{t}^{\prime} } \right)\left( {\frac{{\varepsilon - \varepsilon_{tu} }}{{\varepsilon_{tf} - \varepsilon_{tu} }}} \right)} \hfill & {\varepsilon_{tu} < \varepsilon \le \varepsilon_{tf} } \hfill \\ \end{array} } \right.$$

Here, ${f}_{t}^{\prime}$, ${f}_{cr}$ and ${f}_{tf}$ are the maximum, first cracking and failure tensile strengths of ECC, while ${\varepsilon }_{tu}$, ${\varepsilon }_{cr}$ and ${\varepsilon }_{tf}$ are the corresponding strains, respectively (Fig. 1b). The initial tangent modulus ${E}_{i}$ is taken as $1.5{\left({f}_{c}^{\prime}\right)}^{0.638}$, and ${f}_{t}^{\prime}$ is approximately $0.07{f}_{c}^{\prime}$^23^. The values of ${\varepsilon }_{tu}$, ${\varepsilon }_{cr}$ and ${\varepsilon }_{tf}$ are typically within the ranges 0.6–0.9%, 0.022–0.026% and $50{\varepsilon }_{cr}$, respectively, with lower values corresponding to lower-strength ECC^4,5,9^.

All steel components, including the longitudinal reinforcement, stirrups, and steel section, are modeled using the same constitutive relationship due to their isotropic behavior. In this study, a five-stage constitutive model, illustrated in Fig. 1c, was employed to describe the elastoplastic behavior of steel^26^, as expressed below:

4 $$\sigma_{s} = \left\{ {\begin{array}{*{20}l} {{\text{E}}_{s} \varepsilon_{s} } \hfill & {\varepsilon_{s} < \varepsilon_{e} } \hfill \\ { - A\varepsilon_{s}^{2} + B\varepsilon_{s} + C} \hfill & {\varepsilon_{e} < \varepsilon_{s} \le \varepsilon_{e1} } \hfill \\ {f_{y} } \hfill & {\varepsilon_{e1} < \varepsilon_{s} \le \varepsilon_{e2} } \hfill \\ {f_{y} \left[ {1 + 0.6\frac{{\varepsilon_{s} - \varepsilon_{e2} }}{{\varepsilon_{e3} - \varepsilon_{e2} }}} \right]} \hfill & {\varepsilon_{e2} < \varepsilon_{s} \le \varepsilon_{e3} } \hfill \\ {1.6f_{y} } \hfill & {\varepsilon_{s} > \varepsilon_{e3} } \hfill \\ \end{array} } \right.$$

where ${\varepsilon }_{e}=0.8{f}_{y}/{E}_{s}$, ${\varepsilon }_{e1}=1.5{\varepsilon }_{e}$, ${\varepsilon }_{e2}=10{\varepsilon }_{e1}$, ${\varepsilon }_{e3}=100{\varepsilon }_{e1}$, $A=0.2{f}_{y}/{\left({\varepsilon }_{e1}-{\varepsilon }_{e}\right)}^{2}$, $\text{B}=2\text{A}{\varepsilon }_{e1}$, $\text{C}=0.8{f}_{y}+\text{A}{\varepsilon }_{e}^{2}-\text{B}{\varepsilon }_{e}$ and ${f}_{y}$ and ${E}_{s}$ are the yield stress and elastic modulus of steel.

**Fig. 2:**
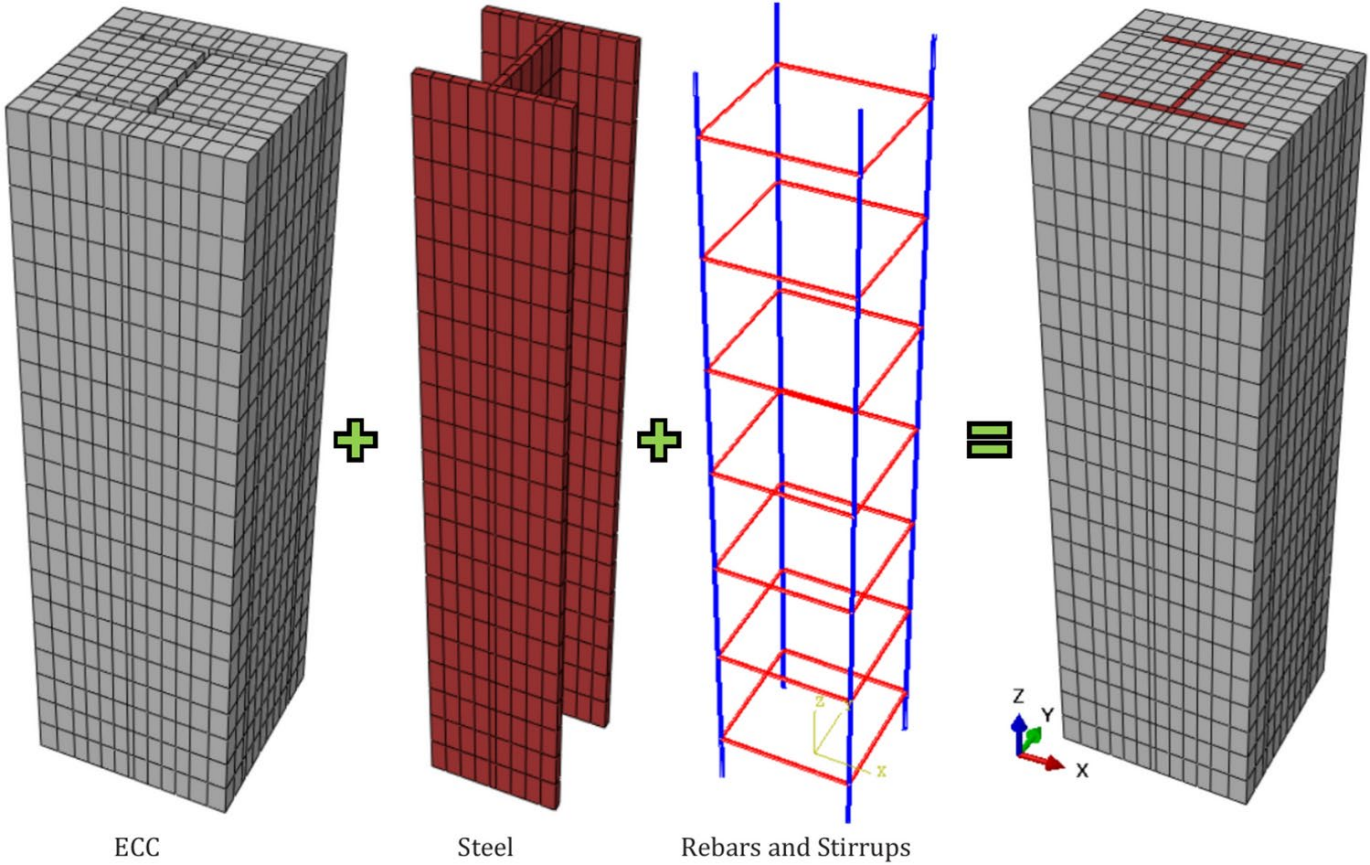
Geometric modelling of ECC-CES columns. The geometric modeling was captured from Abaqus version 6.14^11^.

To ensure accurate predictions, the proposed finite element (FE) model accounted for material and geometric nonlinearities, contact interactions, and geometric imperfections. For the concrete and steel materials, eight-node solid linear brick elements with reduced integration (C3D8R) from the ABAQUS element library were utilized. These C3D8R elements, with three translational degrees of freedom per node, are typically used in nonlinear analyses involving contact between deformable bodies and plasticity under large deformations^11,24^. The longitudinal and transverse reinforcement was represented using T3D2 truss elements, two-node elements embedded throughout the column. A typical view of the ECC-CES column elements is introduced in Fig. 2. A static general solution strategy was employed to analyze the FE model. The top and bottom loading areas were modeled as rigid bodies with reference points. Axial loading was applied via displacement control at the top rigid body reference point. The top of the column was restrained in lateral translational degrees of freedom, allowing only axial deformation in the loading direction. While the bottom support was fully fixed in all translational degrees of freedom.

To further improve prediction accuracy, the FE model incorporated material nonlinearities. For ECC materials, the standard *ELASTIC option in ABAQUS was applied, and the Concrete Damaged Plasticity (CDP) model was used. The CDP model, which is a continuum plasticity-based damage framework, simulates the tensile and compressive behavior of concrete under low confinement pressures^4,11,24^. It requires five major parameters: dilation angle (*ψ*), flow potential eccentricity (*e*), the ratio of biaxial to uniaxial compressive strength (*f*_*b*_*/f’*_*c*_), the ratio of the second stress invariant on the tensile meridian to the compressive meridian (*K*_*c*_), and the viscosity parameter (*v*). In this model, values of (*ψ*, *e*, *f*_*b*_*/f’*_*c*_, *K*_*c*_, and *v*) for ECC were set at (30°, 0.1, 1.17, 0.7, and 0.001), respectively^24^. As this study focuses on the behavior of columns under monotonic loading, the damage variables were not considered^24,27^. For the steel and reinforcement components, the *ELASTIC and *PLASTIC material options from the ABAQUS material library were used to define the elastic-perfectly plastic behavior. For the interaction between steel and ECC, a surface-to-surface cohesive interaction was used^25^. The values of bond strength (τ) and stiffness (K) in normal direction were taken as 100 times the values in shear and tangential direction as described in^25^.

## Verification of finite element model

**Fig. 3:**
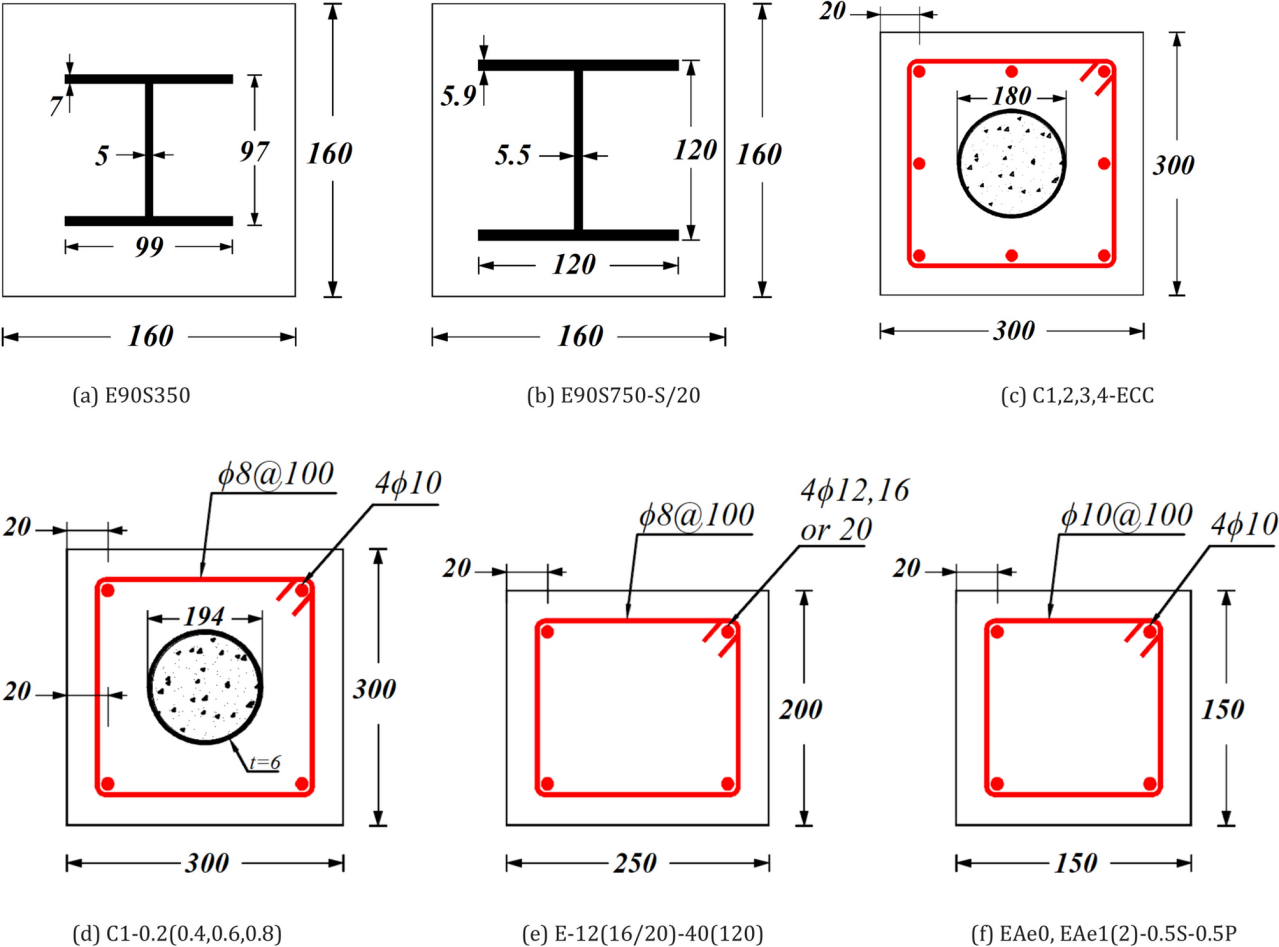
The cross-section geometry of experiment test of CFS columns to be verified.

**Fig. 4:**
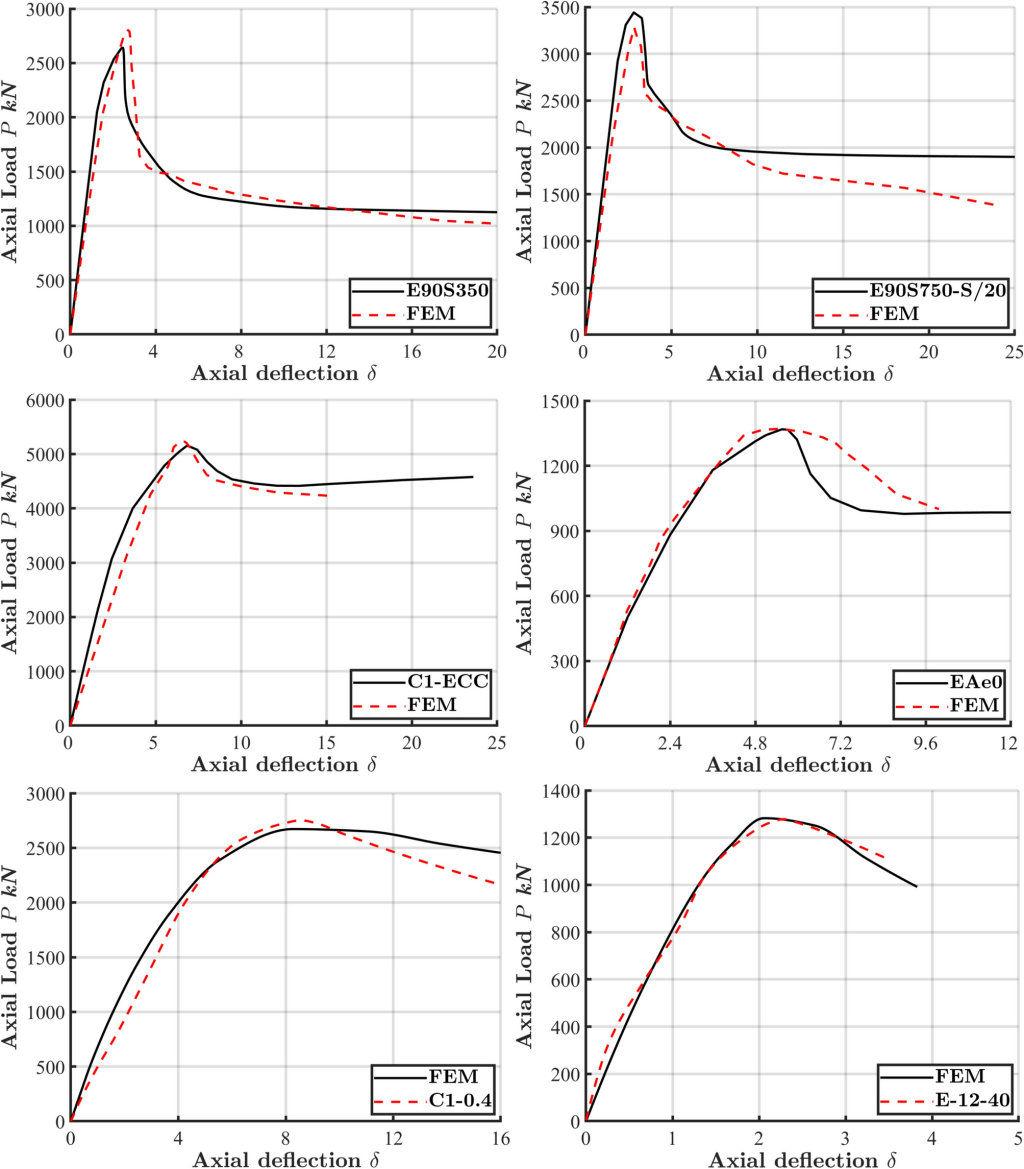

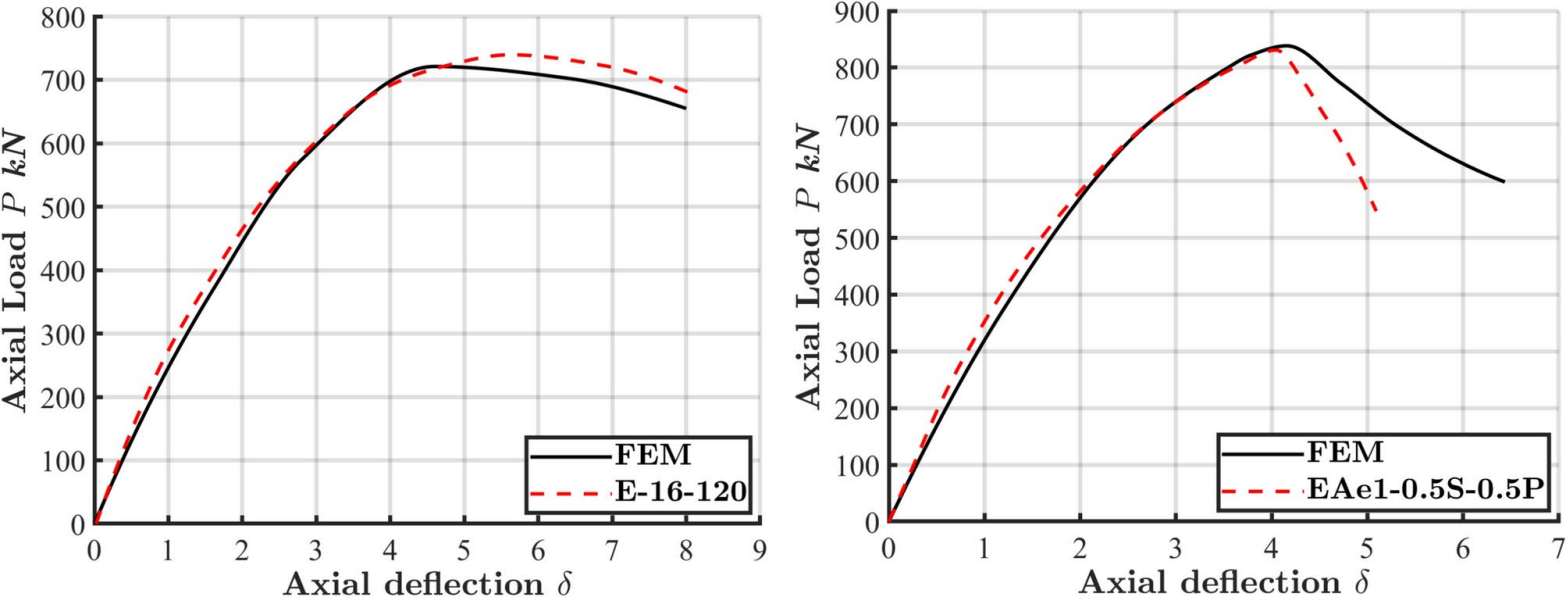
Comparison of load–deflection (L-D) response between experimental and numerical results for specimens E90S350^9^, E90S750-S/20^28^, C1-ECC^29^, EAe0^31^, C1-0.2^31^, E-12-40^32^, E-16-120^32^, and EAe1-0.5S-0.5P^33^.

The accuracy of the FE model in predicting the compressive behavior of ECC-confined concrete was evaluated by comparing the numerical results with experimental data obtained from 19 column specimens, as shown in Table 1 and Fig. 3. Additionally, Fig. 4 illustrates the load–displacement (L-D) curves for eight different column configurations: E90S350^9^, E90S750-S/20^28^, C1-ECC^29,30^, EAe0^31^, C1-0.2^31^, E-12-40^32^, E-16-120^32^, and EAe1-0.5S-0.5P^33^. The model demonstrated reliable accuracy in predicting key structural behaviors, including initial stiffness, peak load, deformation, and post-peak responses. The L-D curves reveal a linear response up to approximately 70% of the peak load, followed by a sharp decline in load-carrying capacity. The sudden drop in strength was more pronounced in columns utilizing higher-strength materials, which aligns with the more brittle nature of high-strength concrete.

**Table 1:** Properties of specimens used for FE validation and comparison of FE peak load predictions with experimental results.

Specimen	Figure [Ref]	Length L	${f}_{c}^{\prime}$	${f}_{y}$	${f}_{r}$	t*	*ρ*_l_**	e (mm)	${P}_{exp}$(kN)	${P}_{FEM}$(kN)	${P}_{FEM}/{P}_{exp}$
E90S350	Figure 3a^9^	500	90.85	377	–	–	–	0	2801	2634	0.94
E90S750-S/20	Figure 3b^28^	500	90.85	752	–	–	–	0	3285	3433	1.045
C1-ECC	Figure 3c^29^	900	32.5	362	358	6	1	0	5197	5143	0.99
C2-ECC						6	1	0	5148	5016	0.974
C3-ECC						10	1	0	6257	5729	0.916
C4-ECC						6	0.5	0	5069	4983	0.983
EAe0	Figure 3f^31^	1200	53.9	–	480	–	1.0	0	1370	1360	0.993
C1-0.2	Figure 3d^31^	1400	32.5	362	358	6	1.0	60	3671	3664	0.998
C1-0.4						6	1.0	120	2768	2752	0.994
C1-0.6						6	1.0	180	1846	1840	0.997
C1-0.8						6	0.9	240	1178	1161	0.986
E-12-40	Figure 3e^32^	1200	46.08	–	534	–	1.6	40	1278	1282	1.003
E-16-40					527	–	2.5	40	1409	1412	1.002
E-20-40					506	–	0.9	40	1572	1569	0.998
E-12-120					534	–	1.6	120	618	600	0.971
E-16-120					527	–	2.5	120	739	721	0.976
E-20-120					506	–	1.4	120	896	870	0.971
EAe1-0.5S-0.5P	Figure 3f^33^	1200	53.9	–	480	–	1.4	30	830	838	1.01
EAe2-0.5S-0.5P						–	1.0	70	515	487	0.946
										Mean	0.984
										CoV	0.028

*t (mm) defines the tube thickness. **ρ_*l*_ (%) stands for the longitudinal reinforcement ratio in Fig. 3c.

NA

Additionally, the peak load predictions by FE model were compared with the experimental peak loads, as shown in Table 1. The FE/test ratios, close to 1.0, indicate a strong correlation between the numerical results and experimental data, particularly regarding column strength. This close alignment between the FE model and test outcomes highlights the model’s accuracy and reliability in simulating the compressive behavior of ECC-CES columns.

**Fig. 5:**
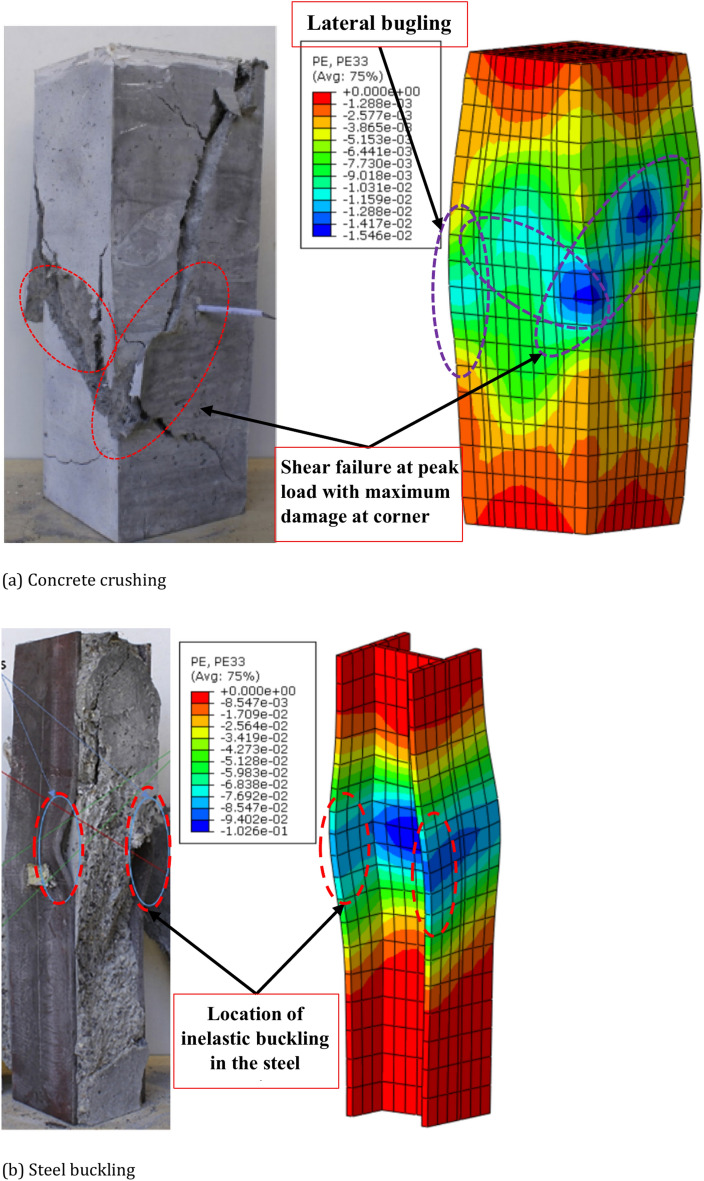
Typical failure mode of column E90S350. The failure mode was captured from Abaqus version 6.14^11^.

Figure 5 presents a comparison between the final-stage failure modes obtained from the FE analysis and experimental results for the E90S350 column. The FE model can successfully predict the column failure modes and provide a reasonable representation of the final damage patterns in terms of plastic strain distribution. Outward bulging of the ECC cover at the mid-height of the column, caused by the lateral dilation of concrete and transverse pushing by the steel, is evident in Fig. 5a. The maximum plastic strain was observed in the areas where the ECC cover experienced the most significant damage, particularly on both the flange and web sides, as well as at the corners. Additionally, plastic strain concentrations were identified in the mid-height regions corresponding to concrete crushing (Fig. 5a) and the inelastic buckling of the steel section (Fig. 5b). The model also accurately captured the plastic damage localized at the corners, which experienced significant shearing under compression (Fig. 5a).

### Bayesian optimization for adaptive experimental design

Design of Experiments (DoE) methods such as factorial design, central composite design, and Latin Hypercube Sampling (LHS)^34^ have been widely adopted to create surrogate models, but these often face challenges in models with high uncertainty or nonlinearity. These traditional methods, while widely used, waste computational resources, especially in high-dimensional problems. Factorial designs for instance, always have size *kd* with level *k* and dimension *d*, making them infeasible choices for problems with many input parameters. Similarly, LHS samples the input space uniformly, which can lead to many evaluations in well-understood regions, making it less efficient in terms of computational cost.

In contrast, Bayesian Optimization (BO) offers a more adaptive approach for experimental design, particularly in complex problems. It works by iteratively refining the surrogate model, typically a Gaussian Process (GP), and selecting new samples based on both the predicted mean and the uncertainty at each iteration. The acquisition function in BO balances exploration (sampling where uncertainty is high) and exploitation (sampling where the predicted mean is promising). This dynamic approach ensures that computational resources are focused on the most informative regions of the design space, making BO more efficient, especially when function evaluations, such as finite element simulations, are costly and limited^14,35^.

Unlike LHS, which does not account for uncertainty in the model, Bayesian Optimization inherently incorporates uncertainty estimation. This allows for targeted exploration of the design space, focusing on regions where the model is uncertain, which is crucial for improving accuracy with fewer simulations. This makes BO particularly effective in scenarios where the objective function is expensive to evaluate, as it reduces the number of simulations required while improving the surrogate model precision.

Bayesian Optimization (BO) consists of two primary components^14,35,36^: a Gaussian process (GP) and an acquisition function. The GP is used to maintain a probabilistic belief over the design space by modeling both the predicted mean ${\mu }_{n}\left({\varvec{x}}\right)$ and the uncertainty ${\sigma }_{n}\left({\varvec{x}}\right)$ at any given point ***x*** in the input space, based on a set of prior observations ${D}_{1:n}=\left\{\left({{\varvec{x}}}_{1},{y}_{1}\right), \left({{\varvec{x}}}_{2},{y}_{2}\right), \dots \left({{\varvec{x}}}_{n},{y}_{n}\right)\right\}$, where ${{\varvec{x}}}_{i}$ represents the input and ${y}_{i}$ is the corresponding output at iteration *i*. This allows for both the prediction of function values at unobserved points and the estimation of uncertainty, which is crucial for determining the next experiment through the acquisition function. The acquisition function selects the most promising point for further exploration by considering both the predicted mean ${\mu }_{n}\left({\varvec{x}}\right)$ and the uncertainty ${\sigma }_{n}\left({\varvec{x}}\right)$.

A GP is fully characterized by its mean function $m\left({\varvec{x}}\right)$ and covariance function $k\left({\varvec{x}},{{\varvec{x}}}^{\prime}\right)$, where the covariance function (also known as the “kernel”) controls the smoothness of the process. Formally, the GP is written as:

5 $$f\left({\varvec{x}}\right)\sim \mathcal{G}\mathcal{P}\left(m\left({\varvec{x}}\right),k\left({\varvec{x}},{{\varvec{x}}}^{\prime}\right)\right)$$

The kernel $k\left({\varvec{x}},{{\varvec{x}}}^{\prime}\right)$ determines the degree of similarity between points in the input space, meaning that when two points ***x*** and ***x****'* are “close” in terms of their inputs, the corresponding outputs *y* and *y'* are expected to be similar. One of the most used kernels is the Gaussian (or Radial Basis Function) kernel, defined as:

6 $$k\left({\mathbf{x}}_{i},{\mathbf{x}}_{j}\right)=\text{exp}\left(-\frac{{\left({{\varvec{x}}}_{i}-{{\varvec{x}}}_{j}\right)}^{T}\left({{\varvec{x}}}_{i}-{{\varvec{x}}}_{j}\right)}{2{l}^{2}}\right)$$

Here, *l* represents the length scale, which controls how quickly the correlation between points decreases with distance. A smaller *l* leads to a faster decrease in correlation.

In real-world applications, observations typically include a noise term, which accounts for measurement errors or system variability. This is modeled by adding normally distributed noise $\epsilon \sim \mathcal{N}\left(0,{\sigma }_{noise}^{2}\right)$ to the process output. Hence, the observation model is given by:

7 $$y= f\left({\varvec{x}}\right)+\epsilon$$

This noise component ensures that the GP can handle noisy data, making BO well-suited for real-world experimental settings where observations are not perfectly accurate.

Gaussian process regression (also known as “kriging”) is a method used to predict the value of the objective function $f\left(\cdot \right)$ at iteration $n+1$ for any location ***x***. The result of this prediction is a normal distribution with a mean ${\mu }_{n}\left({\varvec{x}}\right)$ and uncertainty ${\sigma }_{n}\left({\varvec{x}}\right)$, such that:

8 $$P\left({f}_{n+1}|{D}_{1:n},{\varvec{x}}\right)=\mathcal{N}\left({\mu }_{n}\left({\varvec{x}}\right),{\sigma }_{n}\left({\varvec{x}}\right)\right)$$

where

9 $$\begin{aligned} & \mu_{n} \left( {\varvec{x}} \right) = k^{T} \left[ {K + \sigma_{noise}^{2} I} \right]^{ - 1} y_{1:n} , \\ & \sigma_{n} \left( {\varvec{x}} \right) = k\left( {{\varvec{x}},{\varvec{x}}} \right) - k^{T} \left[ {K + \sigma_{noise}^{2} I} \right]^{ - 1} k \\ \end{aligned}$$

In these equations, *k* represents the covariance vector $\left[k\left({\varvec{x}},{{\varvec{x}}}_{1}\right),k\left({\varvec{x}},{{\varvec{x}}}_{2}\right),\dots ,k\left({\varvec{x}},{{\varvec{x}}}_{n}\right)\right]$, and *K* is the covariance matrix defined as:

10 $$K = \left[ {\begin{array}{*{20}c} {k\left( {{\varvec{x}}_{1} ,{\varvec{x}}_{1} } \right)} & \cdots & {k\left( {{\varvec{x}}_{1} ,{\varvec{x}}_{n} } \right)} \\ \vdots & \ddots & \vdots \\ {k\left( {{\varvec{x}}_{n} ,{\varvec{x}}_{1} } \right)} & \cdots & {k\left( {{\varvec{x}}_{n} ,{\varvec{x}}_{n} } \right)} \\ \end{array} } \right]$$

Once Bayesian Optimization (BO) completes its *k*^th^ iteration, it generates a training dataset ${D}_{k}=\left\{{{\varvec{X}}}_{k}, {{\varvec{y}}}_{k}\right\}$ with *k* samples. The GP model $\left({f}_{k},{\mu }_{k},{\sigma }_{k}^{2}\right)\left({\varvec{x}}\right)$ is developed, and the next step is to determine the most promising point ${{\varvec{x}}}_{k+1}$ for a new finite element (FE) simulation. To minimize the number of simulations, ${{\varvec{x}}}_{k+1}$ is selected based on the information from ${D}_{k}$ and the current GP model $\left({f}_{k},{\mu }_{k},{\sigma }_{k}^{2}\right)\left({\varvec{x}}\right)$. The acquisition function measures the potential of each point in the parameter space to improve the best-observed solution and the next point ${{\varvec{x}}}_{k+1}$ is chosen by maximizing this function:

11 $${\varvec{x}}_{k + 1} = {\text{argmax}}_{{\varvec{x}}} EI\left( {\varvec{x}} \right)\quad \quad {\text{subjected}}\,{\text{to }}{\mathbf{x}} \in \left[ {{\mathbf{x}}_{{\text{l}}} ,{\mathbf{x}}_{{\text{u}}} } \right]$$

where $EI\left(.\right)$ is the acquisition function. A widely used acquisition function, introduced by Jones et al.^1^, is the Expected Improvement:

12 $$EI\left({\varvec{x}}\right)=\left({f}_{min}-\mu \left({\varvec{x}}\right)\right)\Phi \left(\frac{{f}_{min}-\mu \left({\varvec{x}}\right)}{\sigma \left({\varvec{x}}\right)}\right)+\sigma \left({\varvec{x}}\right)\phi \left(\frac{{f}_{min}-\mu \left({\varvec{x}}\right)}{\sigma \left({\varvec{x}}\right)}\right)$$

where Φ(⋅) and *ϕ*(⋅) are the cumulative distribution and probability density functions of the standard normal distribution, respectively, and ${f}_{\text{min}}=\text{min}(\left[f\left({{\varvec{X}}}_{1}\right),\dots ,f\left({{\varvec{X}}}_{k}\right)\right])$. The first term of *EI*(***x***) promotes exploitation by incorporating the best error function value observed so far ${f}_{\text{min}}$, while the second term accounts for exploration by considering the prediction uncertainty $\sigma \left({\varvec{x}}\right)$.

Since the acquisition functions rely primarily on the mean $\mu \left({\varvec{x}}\right)$ and uncertainty $\sigma \left({\varvec{x}}\right)$ of the GP model, it is computationally cheaper to evaluate than the FE model, making BO efficient in selecting the next point for evaluation that maximizes the acquisition function $EI\left({\varvec{x}}\right)$. This approach effectively directs computational resources toward the most impactful areas in the parameter space, reducing the total number of simulations needed.

At each iteration, after selecting a new point ${{\varvec{x}}}_{k+1}$, the GP model and the acquisition function are updated, progressively refining the model by reducing uncertainty ${\sigma }^{2}\left({\varvec{x}}\right)$ across the input domain. This process continues until the uncertainty in the input domain is sufficiently small.

**Fig. 6:**
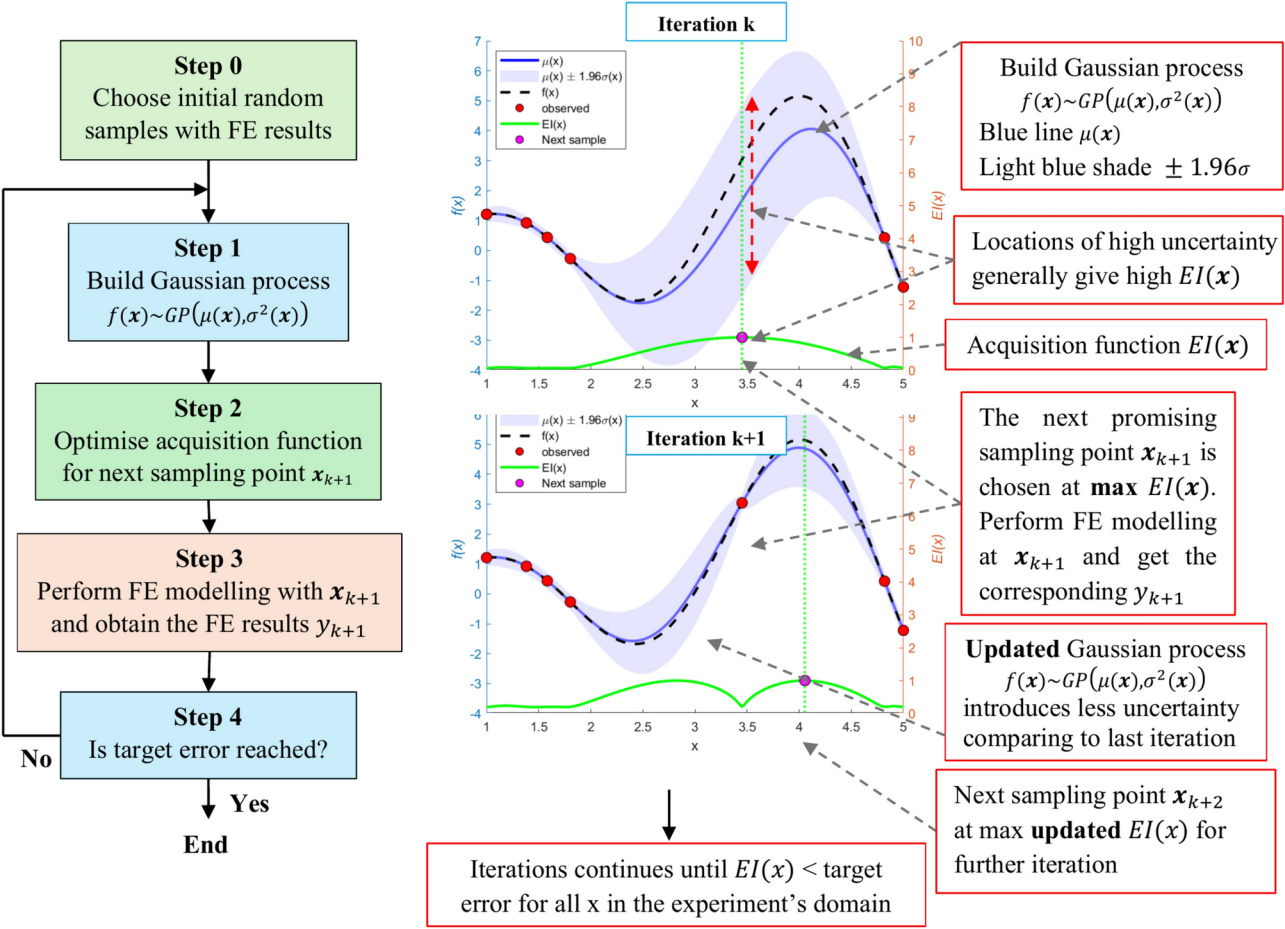
Flow charts of the adaptive sampling.

Figure 6 illustrates the Bayesian optimization flowchart. The right side of the figure shows a GP approximation of the objective function, with the corresponding acquisition function below. In the GP model, $f\left(x\right)\sim GP\left(\mu \left({\varvec{x}}\right),{\sigma }^{2}\left({\varvec{x}}\right)\right)$, each data point ${{\varvec{x}}}_{i}$ has a Gaussian distribution of function values with a mean $\mu \left({{\varvec{x}}}_{i}\right)$ (solid blue line) and variance ${\sigma }^{2}\left({{\varvec{x}}}_{i}\right)$ (light blue shaded region representing the 95% confidence interval). In the initial steps, the surrogate model provides a poor approximation of the true objective function (dashed line). The acquisition function, however, quickly identifies the next point to evaluate, balancing the trade-off between exploitation (low mean) and exploration (high variance). As new training data are added, the GP model is updated (refined), reducing variance and increasing accuracy in approximating the true function. The process stops once a target error, such as root mean square error (RMSE), is achieved across the domain.

### FE database of ECC-CES columns

**Fig. 7:**
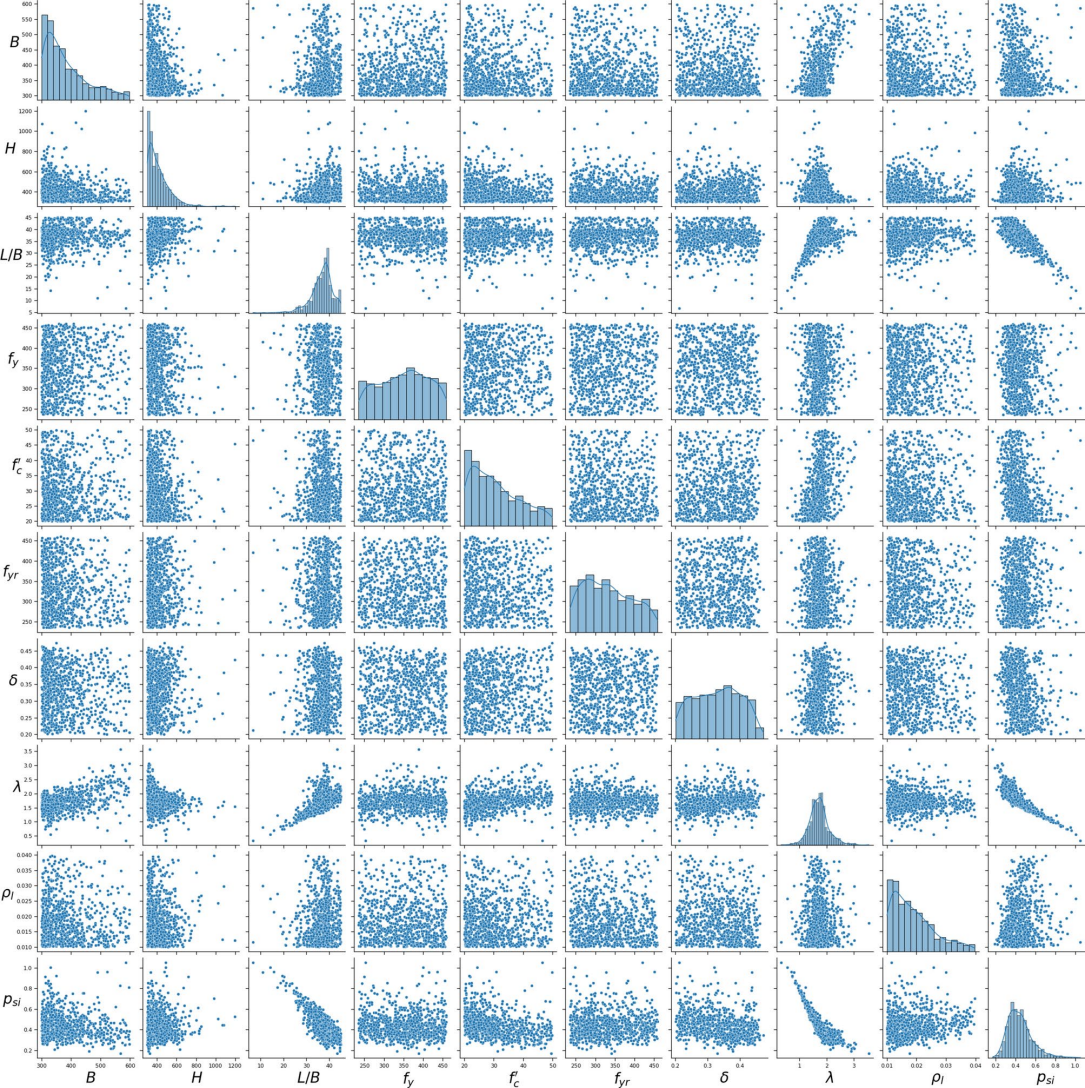
Statistical distribution of the database.

A FE dataset of 840 models was generated using an adaptive sampling process. Based on the results of different experimental and theoretical studies^4,5,9,10^, the axial capacity of ECC-CES columns is influenced by several key components, which include the strength of concrete, steel, and longitudinal reinforcement. Therefore, 11 distinct design features were set as input variables, grouped into four categories: (1) concrete properties: column height (*H*), column width (*B*), Length-to-width ratio (*L/B*), and compressive strength (*f’*_*c*_); (2) steel section properties: steel section height (*h*_*s*_), flange width (*b*_*f*_), web thickness (*t*_*w*_), flange thickness (*t*_*f*_), and yield strength(*f*_*y*_); (4) longitudinal reinforcement properties: reinforcement ratio (*ρ*_*l*_), and yield strength (*f*_*yr*_). The output variable is the dimensionless strength index, denoted as *p*_*si*_. This index is calculated by normalizing the axial load *P*_*u*_ against the combined strengths of the column’s components (steel tubes, core concrete, and longitudinal reinforcement), defined as follows:

13 $$p_{si} = \frac{{P_{u} }}{{N_{pl} }},\quad N_{pl} = A_{r} f_{yr} + A_{s} f_{y} + A_{c} f_{c}^{\prime }$$

where *A*_*s*_, *A*_*r*_ and *A*_*c*_ are the steel, longitudinal reinforcement, and concrete areas, respectively. Table 2 and Fig. 7 summarize statistical information for both the output and the 11 input features from the established database. The strength index, *p*_*si*_, reflects global column slenderness, as a relatively slender column (characterized by a larger *L/B* ratio) results in a lower strength index value. It was found that using the strength index (*p*_*si*_) instead of axial column capacity (*P*_*u*_) as the main output enhances the ML prediction performance^37,38^ as the statistical distribution of the strength index closely resembles a normal distribution. This is illustrated in Table 2, where *p*_*si*_ has a skewness of 1.19, compared to the much higher skewness of 4.51 for *P*_*u*_. It is important to acknowledge that the parameter ranges of ECC-CES samples in the databases fall inside the scope of EC4 design code^39^, as illustrated in Table 2 and Fig. 7.

**Table 2:** Statistic features of the FE dataset.

Variable	Symbol	Statistics					
		Min	Max	Mean	Std	Skewness	Kurtosis
Column width	$B$ (mm)	300	600	383.3	73.33	1.07	0.31
Column height	$H$ (mm)	300	1200	416.6	113.9	2.08	7.15
Length-to-width ratio	$L/B$	5	45	36.7	4.89	− 1.3	3.85
Reinforcement yield strength	${f}_{yr}$ (MPa)	235	460	332.9	61.4	0.29	− 1.02
Concrete compressive strength	${f}_{c}^{\prime}$ (MPa)	20	50	30.8	8.08	0.62	− 0.65
Steel section yield strength	${f}_{y}$ (MPa)	235	460	350.9	62.84	− 0.1	− 1.08
Steel contribution ratio	$\delta$*	0.2	0.47	0.33	0.07	− 0.05	− 1.12
Web thickness	${t}_{w}$ (mm)	1.65	30.0	7.74	3.94	1.39	2.86
Flange thickness	${t}_{f}$ (mm)	2.59	62.0	11.37	5.94	2.09	8.95
Steel section height	${h}_{s}$ (mm)	103.6	1105	275.5	119.6	1.92	6.66
Flange width	${b}_{f}$ (mm)	107.7	512.3	238.6	81.4	0.83	0.2
Slenderness ratio	$\overline{\lambda }$**	0.34	3.56	1.72	0.357	0.54	2.06
Longitudinal reinforcement ratio	${\rho }_{l}$	0.01	0.04	0.019	0.007	0.93	0.167
Axial load	${P}_{u}$ (N)	1205	18,709	3062	1366	4.51	36.83
Normalized load	${p}_{si}$***	0.165	1.049	0.435	0.124	1.19	2.314

*$\delta =\frac{{A}_{s}{f}_{y}}{{\text{A}}_{\text{s}}{f}_{y}+{A}_{r}{f}_{y}+{A}_{c}{f}_{c}^{\prime}}$ is the steel contribution ratio, where *A*_*s*_, *A*_*r*_, and *A*_*c*_ are the steel, rebar, and concrete areas, respectively.

**Slenderness ratio $\overline{\lambda }$ is defined in Table 3 for EC4^39^.

***${p}_{si}=\frac{{P}_{u}}{{\text{A}}_{\text{s}}{f}_{y}+{A}_{r}{f}_{y}+{A}_{c}{f}_{c}^{\prime}}$ is the normalized load.

### ML algorithms

This study employs seven machine learning (ML) models for the axial strength of ECC-CES columns, including the Gaussian process (GPR)^40^, light gradient-boosting machine (LGBM)^41^, random forests^42^, categorical boosting (CATB)^43^, extreme gradient boosting (XGBoost)^44^, support vector regression^45^, and symbolic regression (SR)^46,47^. The performance of these models is evaluated and compared. In general, ensemble learning techniques often yield greater accuracy and stability than individual models^43^.

CATB, LGBM, and XGBoost are ensemble learning techniques based on boosting, where weak learners are iteratively combined into a stronger predictor^48^. CATB is especially efficient with categorical features and eliminates the need for preprocessing non-numerical features^43^. By using unbiased boosting, it reduces gradient bias and improves generalization ability when handling categorical data. LGBM^41^ utilizes a histogram-based approach for data splitting, which accelerates training and is advantageous for large datasets. XGBoost^44^, in contrast, adopts a level-wise depth-first approach, which can be slower than LGBM but may offer more robust solutions for specific tasks. On the other hand, Random forests, proposed by Breiman^42^, is an ensemble learning method that uses bagging. It creates subsets of data to train weak learners, such as decision trees, and combines their outputs through averaging (for regression) or voting (for classification). Several key parameters, such as the number of trees, the maximum number of features, and the maximum depth of the trees, influence the performance of the model.

Symbolic regression (SR)^46,47^ based on genetic programming, seeks to derive interpretable mathematical formulas that balance accuracy and complexity. SR operates by generating tree-like expressions and refining them through principles of natural selection. PySR library in Python^49^ is employed in this research to predict the axial capacity of ECC-CES columns. The SR process involves creating a population of expressions, selecting top performers, and applying mutations and crossovers to evolve new formulas. A fitness function ensures a balance between model accuracy and simplicity, avoiding unnecessary complexity in the generated expressions. The proposed design model derived from SR is presented in Table 3.

**Table 3:** Summary of code standards and proposed design in predicting axial strength of ECC-CES columns.

	Formulas
AISC360^54^	$${P}_{AISC}=\left\{\begin{array}{c}{P}_{no}\left[{0.658}^{\frac{{P}_{no}}{{P}_{e}}}\right], \frac{{P}_{no}}{{P}_{e}}\le 2.25\\ 0.877{P}_{e}, \frac{{P}_{no}}{{P}_{e}}>2.25\end{array}\right.$$ ${P}_{e}=\frac{\pi E{I}_{eff}}{{\left(KL\right)}^{2}}$*,*$E{I}_{eff}={E}_{s}{I}_{s}+0.5{E}_{s}{I}_{sr}+{C}_{1}{E}_{c}{I}_{c}, {C}_{1}=0.1+2\left(\frac{{A}_{s}}{{A}_{c}+{A}_{s}}\right)\le 0.3$ ${P}_{no}={A}_{s}{f}_{y}+{A}_{r}{f}_{yr}+0.85{A}_{c}{f}_{c}$
EC4^39^	${P}_{EC4}= \chi {P}_{no}$ $\chi =\frac{1}{\Phi +\sqrt{{\Phi }^{2}-{\overline{\lambda }}^{2}}} , \Phi =0.5\left[1+\alpha \left(\overline{\lambda }-{\overline{\lambda }}_{0}\right)+{\overline{\lambda }}^{2}\right]$ ${P}_{no}={A}_{s}{f}_{y}+{A}_{r}{f}_{yr}+0.85{A}_{c}{f}_{c}$ $\overline{\lambda }=\sqrt{\frac{{P}_{no}}{{N}_{cr}}}, {N}_{cr}=\frac{{\pi }^{2}{\left(EI\right)}_{eff}}{{L}_{e}^{2}}, {\left(EI\right)}_{eff}={E}_{a}{I}_{a}+{E}_{s}{I}_{s}+0.6{E}_{c}{I}_{c}$
Proposed design	${P}_{EC4}=\chi {P}_{no}$ $$\chi =\left\{\begin{array}{c}1.0, \overline{\lambda }<0.67\\ \beta -\sqrt{{\beta }^{2}-\frac{1}{0.64\overline{\lambda }}}, \overline{\lambda }\ge 0.67\end{array}\right. , \beta =0.16+0.25\overline{\lambda }+\frac{0.9}{\overline{\lambda }}$$ $$\begin{aligned}{P}_{no}&={A}_{a}{f}_{y}+{A}_{r}{f}_{yr}+0.8{A}_{c}{f}_{c}, \overline{\lambda }=\sqrt{\frac{{P}_{no}}{{N}_{cr}}}, {N}_{cr}\\&=\frac{{\pi }^{2}{\left(EI\right)}_{eff}}{{\left(KL\right)}^{2}}, {\left(EI\right)}_{eff}=\text{min}\left(E{I}_{x},E{I}_{y}\right),\end{aligned}$$ $E{I}_{x}={E}_{a}{I}_{a}+{E}_{s}{I}_{s}+{\alpha }_{x}{E}_{c}{I}_{c}, {\alpha }_{x}=0.8-0.75\delta ,$ $E{I}_{y}={E}_{a}{I}_{a}+{E}_{s}{I}_{s}+{\alpha }_{y}{E}_{c}{I}_{c}, {\alpha }_{y}=0.5-0.55\delta ,$ $\delta ={A}_{s}{f}_{y}/({A}_{a}{f}_{y}+{A}_{r}{f}_{yr}+{A}_{c}{f}_{c}), 0.2\le \delta \le 0.47$

### Data preprocessing and hyperparameter Bayesian optimization technique

In this study, the min–max scaling technique is employed for data normalization to mitigate the challenges posed by multidimensionality. After normalization, the dataset is divided into two subsets: 80% of the data is randomly assigned to the training set, while the remaining 20% is reserved for testing.

The performance of most machine learning (ML) algorithms is highly dependent on their hyperparameters, which must be defined before model training. Proper tuning of these hyperparameters is crucial for achieving optimal predictive performance. Finding the best hyperparameters requires exploring different combinations and selecting the set that performs best on the validation data. Traditional methods like grid search (GS) and random search (RS) can be exhaustive and time-consuming, especially when dealing with models with numerous hyperparameters and large search spaces. In contrast, Bayesian Optimization (BO) models employ surrogate functions, such as Gaussian processes and tree-structured Parzen estimators(TPE)^50^, to guide the selection of the next hyperparameter set based on the performance of previously tested combinations. This approach reduces redundant evaluations, allowing BO to identify the optimal hyperparameter combination in fewer iterations compared to GS and RS methods^51^. In this study, the TPE model^50^ was adopted to optimize the hyperparameters of the ML models due to its superior robustness compared to other surrogate functions^51^.

### Performance and results of ML models

**Fig. 8:**
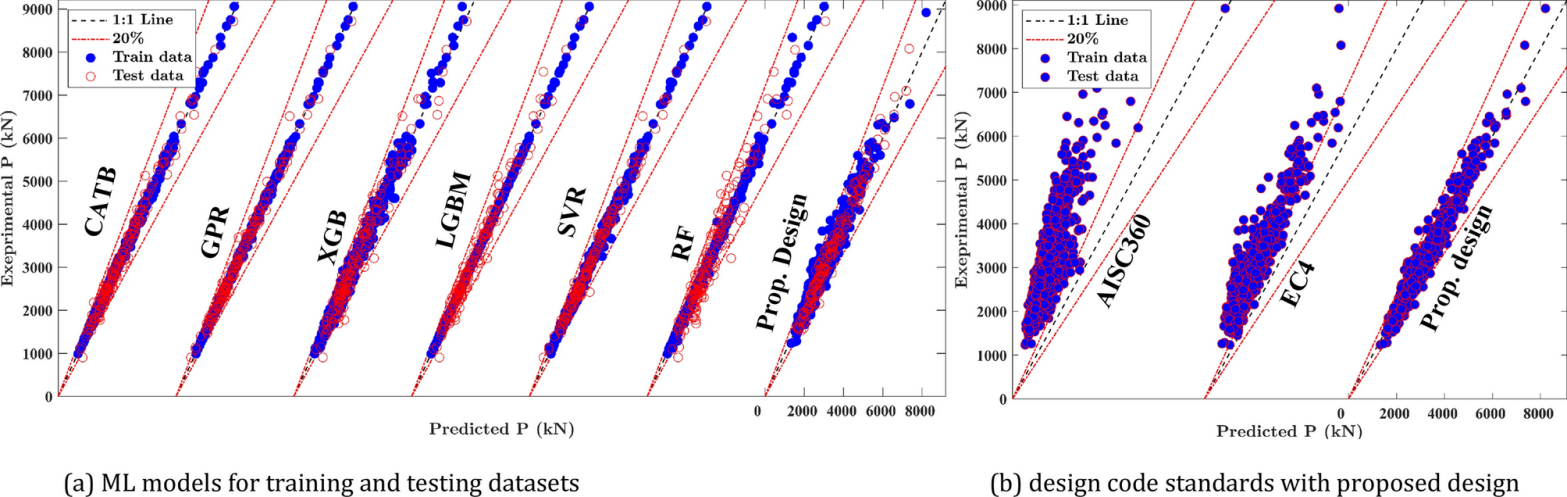
Prediction results of ML models.

In this section, the performance comparison of the introduced ML models is explained. The details of developed ML models are provided in supplementary data, including hyperparameter tuning and results. The scatter plots in Fig. 8a show the relationship between experimental and predicted outcomes for various machine learning (ML) models applied to both the training and testing datasets for ECC-CES columns. For most models, the data points cluster closely around the diagonal line, which indicates a strong alignment between the model predictions and the experimental results. This alignment highlights the reliability and accuracy of the developed ML models. Table 4 presents several evaluation metrics used to assess the performance of these ML models. These metrics include: (1) mean (*μ*): measures the ratio between actual and predicted values, (2) coefficient of variance (CoV): assesses the variability relative to the mean, (3) coefficient of determination (*R*^*2*^): indicates the proportion of variance in the dependent variable explained by the model, (4) root mean squared error (RMSE): quantifies the average prediction error, (5) the mean absolute percentage error (MAPE): evaluates the percentage error between predicted and actual values, and (6) a20-index^52,53^: represents the percentage of predictions where the ratio of actual to predicted values falls within 0.80 to 1.20. The formulas for these metrics are provided as follows:

14 $$\mu = \frac{1}{n}\mathop \sum \limits_{i = 1}^{n} \frac{{y_{i} }}{{\hat{y}_{i} }}, \quad R^{2} = 1 - \frac{{\mathop \sum \nolimits_{i = 1}^{n} \left( {\hat{y}_{i} - y_{i} } \right)^{2} }}{{\mathop \sum \nolimits_{i = 1}^{n} \left( {y_{i} - \overline{y}} \right)^{2} }} \quad RMSE = \sqrt {\frac{1}{n}\mathop \sum \limits_{i = 1}^{n} \left( {\hat{y}_{i} - y_{i} } \right)^{2} } ,\quad MAPE = \frac{1}{n}\mathop \sum \limits_{i = 1}^{n} \left| {\frac{{y_{i} }}{{\hat{y}_{i} }} - 1} \right| \times 100\%$$

where ${y}_{i}$ and ${\widehat{y}}_{i}$ are the actual FE and predicted output values of the *i-*th sample, respectively, $\overline{y }$ is the mean value of FE output results, and *n* is the number of FE specimens in the database.

**Table 4:** Comparison of the developed ML models.

Metrics	Training data			Testing data			All data					
	GPR	CatB	LGBM	GPR	CatB	LGBM	GPR	CatB	LGBM	AISC^54^	EC4^39^	Prop. Design
Mean $\mu$	1.00	0.999	1.00	1.003	0.997	0.993	1.00	0.999	0.999	2.129	1.423	1.007
CoV	0.014	0.019	0.004	0.045	0.056	0.065	0.024	0.03	0.029	0.184	0.171	0.069
R^2^	0.999	0.998	1.00	0.995	0.989	0.986	0.998	0.995	0.996	0.779	0.918	0.974
MAPE%	1.073	1.515	0.267	2.972	4.214	4.594	1.452	2.055	1.133	112.911	42.334	5.221
RMSE (kN)	43.1	57.7	11.3	120.2	191.3	206.1	66.2	99.9	92.7	1681.7	952.1	227.5
a20-index	1.00	1.00	1.00	0.994	0.994	0.994	0.999	0.999	0.999	0.012	0.079	0.987

The evaluation metrics in Table 4 show that all the ML models display mean *μ*, *R*^*2*^, and a20-index values close to 1.0, along with small values for CoV, MAPE%, and RMSE. The prediction results of all introduced models exhibit CoV less than 0.07, and MAPE% lower than 5% for testing subset, indicating minimized scattering in the prediction results compared to the experimental results. As shown in Table 4, the Gaussian Process Regression (GPR) model shows the best performance, achieving the lowest MAPE% values of 1.07% for the training set and 2.97% for the testing set. Similarly, those of the CatBoost (CATB) model are 1.52% and 4.21%, and those of the LightGBM (LGBM) model are 0.27% and 4.59%, indicating the high accuracy of the developed models. Although the LGBM model exhibits slightly higher errors for the testing data, achieving a MAPE% of 4.59 compared to the training set error with a MAPE% of 0.27, its overall performance, as measured by remaining evolution metrics, is comparable to other ML models. Furthermore, the *μ* values of the GPR model are 1.00 and 1.003, the *R*^*2*^ values are 0.999 and 0.995, and the a20-index values are 1.00 and 0.994 in the training and testing sets, respectively, which are close to 1.00. Such evaluation metrics reveal that the GPR model introduces the best prediction accuracy and predictive balance between the training and testing sets.

While the GPR, CATB, and LGBM models demonstrate superior predictive accuracy, their black-box nature poses challenges for practical implementation in engineering design. These models are difficult to interpret and derive explicit design formulas from, which limits their usability in real-world applications. In contrast, the Symbolic Regression (SR) algorithm offers a distinct advantage by generating simple, interpretable, and practical explicit design formulas. As shown in Table 4, the proposed design extracted from the SR algorithm yields a mean of *μ* of 1.007, an *R*^*2*^ of 0.974, and a CoV of 0.069. Although these metrics show a slightly lower accuracy than the introduced ML models, the SR-derived design in Table 3 is much easier to interpret, facilitating their practical use in engineering contexts.

The compressive strength predictions of ECC-CES columns using the proposed SR-derived equations were compared with the existing design code formulas, including EC4^39^ and AISC360^54^. Although no specific design standards exist for ECC-CES columns, numerous researchers have observed that code-based predictions tend to be conservative, often underestimating the actual strength when compared to experimental results^4,5,9,10^. Table 4 reveals that the proposed SR-derived equations achieve performance metrics with a mean, R2, and a20-index near 1.0, a CoV of 0.069, and a MAPE% of 5.22%. Meanwhile, the EC4 and AISC360 code formulas demonstrate much larger errors, with CoV values of 0.171 and 0.184, and MAPE% values of 42% and 113%, respectively. In addition, the AISC360^54^ and EC4^39^ predictions, compared to proposed design predictions, appear to underestimate the axial capacity with a mean *μ* approaching 1.42 and 2.13, very small a20-index, and relatively high error indices. This is further supported by the scatter plots in Fig. 8b, which show that both AISC360 and EC4 exhibit over-diagonal skewed distributions, indicating their conservative nature in predicting axial capacity. The RMSE and MAPE% values of AISC360 and EC4 design codes are approximately four to seven times higher than those produced by the proposed SR equations, suggesting that relying on these design standards directly may result in overly conservative estimates, particularly for columns with relatively high slenderness ratios. Although the design standards ensure safe designs, the evaluation metrics introduced by the ML models and the SR-derived formulas reveal significantly smaller error indices than the conventional design standards. Specifically, the proposed equations demonstrate superior performance across all evaluation criteria, making them a valuable tool for practical and accurate prediction of column behavior.

**Fig. 9:**
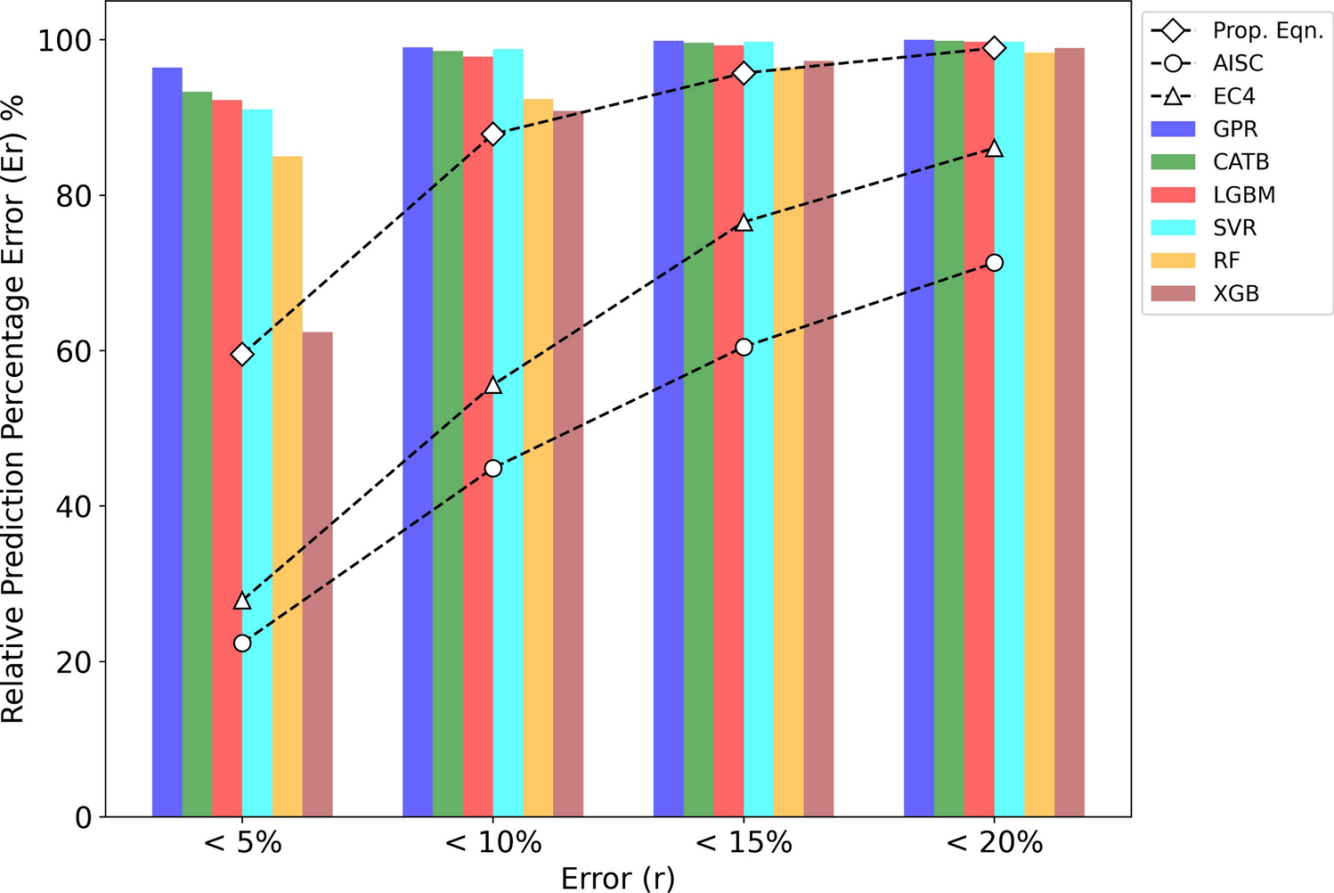
Prediction errors of design standards and established ML models.

Figure 9 illustrates the prediction errors for both the design standards and the introduced ML models. Due to relatively large mean values $\mu$ for design codes, their prediction errors are nearly zero. However, the mean value *μ* alone does not fully capture the quality of predictions; instead, the uncertainty associated with predictions, such as the CoV, provides a better measure of prediction reliability. For example, although the mean value *μ* of AISC360 is approximately 1.5 times greater than that of EC4, both codes display comparable prediction errors due to their similar CoV values, as shown in Table 4. The difference in mean values *μ* can be addressed through reliability analysis and the adjusting safety factors. Therefore, a new prediction error metric, named the Relative Prediction Percentage Error (Er), is introduced. This metric is not influenced by the mean *μ* and is defined as:

15 $${E}_{r}=\frac{1}{n}\sum_{i=1}^{n}H\left(\left|\frac{{y}_{i}}{\mu {\widehat{y}}_{i}}-1\right|\times 100\%-r\right),$$

where *r* is the error range percentage $r\in \left[0-100\%\right]$, $\mu$ is the mean defined previously, $H\left(x-r\right)$ is the unit step function defined as:

16 $$H\left( {x - r} \right) = \left\{ {\begin{array}{*{20}c} {1,\quad x < r} \\ {0,\quad x \ge r} \\ \end{array} } \right., \mu = \frac{1}{n}\mathop \sum \limits_{i = 1}^{n} \frac{{y_{i} }}{{\hat{y}_{i} }}$$

Figure 9 reveals that most of the introduced ML models surpass the design standards in terms of accuracy. Specifically, GPR, CATB, and LGBM models show more than 97% of FE samples within the 10% error range (${E}_{10\%}>97\%)$. Meanwhile, the proposed designs, EC4 and AISC360, show 88%, 59%, and 40% of FE samples, respectively, within the same range. These results highlight the superior accuracy of the introduced ML models, particularly GPR, CATB, and LGBM, in predicting the axial strength of ECC-CES columns compared to traditional design standards. Furthermore, the proposed design outperforms both EC4 and AISC360, as shown in Fig. 9, with nearly 1.5 times and twice as many test samples within the same error range compared to EC4 and AISC360, respectively. Thus, the introduced ML models can be considered valuable tools for estimating the axial capacity of ECC-CES columns, complementing traditional design standards and providing enhanced predictive accuracy.

## Limitations and future research directions

This section highlights the limitations of the developed data-driven models and explores potential directions for future research. The validity of the proposed ML models is limited to the range of input parameter values specified in Table 2, which defines the scope of their applicability for reliable predictions. The accuracy of the models may diminish when applied to extreme input values or novel column configurations beyond the range of the training data. Future research could focus on strategies to improve the models’ generalization capabilities for these scenarios. In addition, the introduced FEA model incorporates material nonlinearity, confinement effects, and realistic boundary conditions, which inherently capture some aspects of size-dependent behavior. However, the size effect in shear and other failure mechanisms might not be fully represented without explicit size-scaling factors or advanced techniques like nonlocal or gradient-based modeling. For real-world applications where column dimensions exceed the tested range, we recommend applying size-effect correction factors based on experimental or analytical studies. Additionally, future work could include extending the dataset to larger specimen sizes (e.g., 600–2000 mm) to further validate the model’s scalability. Furthermore, the present study is centered on columns subjected to axial loading. Future work could expand the analysis to encompass other loading conditions, such as eccentric or biaxial eccentric loading scenarios, providing a more comprehensive understanding of ECC-CES column behavior. Furthermore, exploring the application of the SR model to other types of composite columns, such as double-skin CFST or hybrid composite columns (see for example Fig. 3c), could broaden its applicability.

Future investigations into ECC columns could delve into the use of ECC encasement as an alternative to traditional stirrups for confining conventional concrete, potentially simplifying construction processes and improving structural performance^9^. The behavior of column-beam connections incorporating ECC encasement is another promising area of study, particularly for understanding its impact on structural integrity and seismic resilience. Additionally, exploiting ECC’s unique properties—such as high resistance to climatic conditions, chemical exposure, and corrosion—could facilitate the development of more durable and sustainable structural systems^6–8^. ECC’s superior energy dissipation, damage absorption, and crack resistance compared to conventional concrete also present opportunities to enhance the performance, ductility, and energy absorption capacity of structural elements under diverse loading conditions. In addition, due to the relatively high cost of ECC compared to conventional concrete, ML techniques can be utilized to optimize its application, ensuring cost-effectiveness while maximizing its structural advantages. These research directions can pave the way for the broader adoption of ML techniques for ECC in advanced structural engineering applications.

### Research significance

This research advances the modeling of ECC-CES columns by integrating finite element analysis with adaptive sampling, generating a comprehensive FE database. By leveraging advanced machine learning techniques, the study enhances prediction accuracy, outperforming traditional design standards. The findings offer valuable insights for developing more efficient, reliable structural design methods.

## Conclusion

This study developed a 3D nonlinear finite element model for analyzing ECC-CES columns, accounting for both material and geometric nonlinearities. An adaptive sampling process was utilized to generate 840 finite element (FE) models, allowing for efficient design space exploration. Additionally, seven widely used machine learning (ML) models were employed, and their performance was rigorously evaluated and compared. The following conclusions can be made:

1. The FE model demonstrated a high level of accuracy in predicting the compressive behavior of ECC-CES columns, with a strong correlation between the numerical results and experimental data. The FE/test ratios were consistently close to 1.0, validating the model’s reliability.
2. Adaptive sampling significantly enhances the accuracy of surrogate models used for column strength prediction by focusing computational resources on areas with high uncertainty or significant impact. This approach reduces the number of simulations needed while ensuring comprehensive model performance across the entire input domain.
3. The developed machine learning (ML) models, particularly the GPR, CATB, and LGBM models, demonstrated superior predictive accuracy in estimating the axial capacity of ECC-CES columns compared to traditional design standards such as EC4 and AISC360.
4. While black-box ML models are challenging to interpret, the symbolic regression (SR) approach provides explicit design formulas that are simple, interpretable, and practically useful in engineering applications.
5. The proposed SR-based equations performed better than existing code formulas, achieving nearly identical mean values (*μ* ≈ 1.00), low CoV, and high R^2^, making them competitive alternatives to both ML models and design codes in terms of accuracy and ease of implementation.
6. Traditional design standards, such as EC4 and AISC360, were found to overestimate axial capacity with high errors. These standards displayed relatively large prediction errors (RMSE, MAPE) compared to the proposed ML models and SR equations.
7. A novel metric, the relative prediction percentage error (*E*_*r*_), was introduced to assess prediction quality by focusing on variability (CoV) rather than mean value (*μ*). Over 97% of the samples predicted by GPR, CATB, and LGBM models fall within a 10% error range, highlighting their reliability and accuracy.

The study highlights the potential of ML models to complement existing design standards. The superior accuracy of ML models, particularly in cases where design codes are conservative, shows that ML can be a valuable tool for refining and validating design calculations in engineering practice.

## Supplementary Information

Supplementary Information.

## Data Availability

All data generated or analyzed during this study are included in this published article and available in a public repository https://github.com/kmegahed/ECC-CES-columns.
